# Longitudinal Studies of Sleep Disturbances in Parkinson’s Disease

**DOI:** 10.1007/s11910-022-01223-5

**Published:** 2022-08-26

**Authors:** Zheyu Xu, Kirstie N. Anderson, Nicola Pavese

**Affiliations:** 1 National Neuroscience Institute, Singapore, Singapore; 2 Regional Sleep Service, Newcastle Upon Tyne NHS Foundation Trust, Newcastle upon Tyne, UK; 3 Institute of Neuroscience, Newcastle University, Newcastle upon Tyne, UK; 4 Department of Nuclear Medicine and PET Centre, Aarhus University Hospital, Aarhus, Denmark; 5 Newcastle Magnetic Resonance Centre, Newcastle University, Campus for Ageing & Vitality, Westgate Road, Newcastle upon Type, NE4 5PL UK

**Keywords:** Parkinson’s disease (PD), Sleep disorders, Longitudinal studies, Rapid eye movement sleep behavior disorder (RBD), Excessive daytime sleepiness (EDS)

## Abstract

**Purpose of Review:**

Sleep disorders are among the most common non-motor symptoms in Parkinson’s disease (PD). Recent longitudinal studies of sleep in PD have utilized validated sleep questionnaires and video-polysomnography performed over multiple time points. This review summarizes existing longitudinal studies focusing on the prevalence, associations, and changes of sleep disorders in PD over time, as well as the methodologies used in these studies.

**Recent Findings:**

Fifty-three longitudinal studies of sleep in PD were identified: excessive daytime sleepiness, insomnia, obstructive sleep apnea, rapid eye movement sleep behavior disorder (RBD), restless legs syndrome, and shift work disorder were studied in addition to other studies that had focused on either multiple sleep disorders or broadly on sleep disorders as a whole. The prevalence of sleep disorders increases over time and are associated particularly with non-motor features of disease. RBD is now considered an established prodromal feature of PD, but other sleep disorders do not clearly increase risk of subsequent PD. Further work is necessary to determine if treatment of sleep disorders in PD alters disease symptom and their progression or reduces PD risk.

**Summary:**

Longitudinal studies of sleep in PD have demonstrated a high prevalence of sleep disorders that are associated with non-motor features of PD which can increase over time. More work is necessary to determine if treatment of sleep disorders can alter the course of PD.

## Introduction

Sleep disturbances are one of the most common non-motor symptoms in Parkinson’s disease (PD). Although sleep disturbances are typically a feature of advanced disease, they are also recognized to be present both at the prodromal and early stages of disease [1]. A broad range of sleep disorders are seen in PD including rapid eye movement (REM), sleep behavior disorder (RBD), excessive daytime sleepiness (EDS), insomnia, restless legs syndrome (RLS), and sleep disordered breathing. Cross-sectional studies studying a range of sleep disorders in PD report a high prevalence of any sleep disorders that exceed 95% [2] [3]. RBD has been of particular research interest as the disorder is now recognized as a prodromal feature of PD and other Parkinsonisms and can precede the development of these conditions by up to 20 years.

In 2011, Zoccolella and colleagues analyzed 17 longitudinal studies and found that the prevalence of sleep disorders increases with disease duration, and that its presence could be indicative of a more aggressive disease course or associated with particular phenotypes within PD [4]. Since then, there has been a growing interest in the study of sleep in PD: recommendations for PD sleep questionnaires have been published [5], further longitudinal PD cohorts studied, and gold standard video polysomnography (vPSG) utilized in longitudinal studies to better characterize sleep. The purpose of this review is to critically review existing longitudinal studies reporting on prevalence, associations, and changes over time of sleep disorders in PD. We have also examined the relationship between specific sleep disorders and future risk of PD. Finally, we have identified limitations in existing methodologies. We have identified 53 longitudinal studies of sleep in PD from 1996 which are summarized in Table 1.

**Table 1:** Longitudinal studies of sleep in Parkinson’s disease

Authors (country)	Study design/duration of follow-up	Study population	Sleep diagnostic procedures and rating scales	Results
Excessive daytime sleepiness				
[6] UK	Prospective cohort, 4.8 ± 1.98 years	923 early PD (diagnosed within 3.5 years)	ESS	ESS scores in PD increased from baseline 7.6 ± 4.5 to 8.7 ± 4.77 during the period of follow-up
[7] Sweden	Prospective cohort, 10 years	30 moderate disease PD	ESS	ESS scores in PD remained stable during the period of follow-up At an individual level, the degree of EDS as measured using the ESS could vary over time EDS was associated with sleep quality, fatigue, anxiety, depression, and axial/postural/gait impairments
[8] PPMI cohort	Prospective cohort, 3 years	423 early disease, de novo PD subjects and 196 HCs	ESS	ESS scores in PD increased from baseline to 3 years (mean ± SD 5.8 ± 3.5 to 7.55 ± 4.6, *p* < 0.0001) No change in ESS scores were observed in HCs EDS in PD was associated with the non-tremor dominant phenotype, autonomic dysfunction, depression, anxiety, and probable behavior disorder, but not cognitive dysfunction or motor severity Dopaminergic therapy was associated with EDS at years 2 and 3 EDS was associated with presynaptic dopaminergic dysfunction on the DAT scan but not CSF biofluid markers
[9, 10] Norway	Population based cohort, 5 years	153 drug naïve, early PD patients, 169 HCs	ESS	The frequency of EDS in PD increased from 11.8% at baseline to 23.4% after 5 years; PD patients reported more EDS than control participants at all time points Increased baseline ESS scores predicted the subsequent development of EDS The presence of EDS was associated with the male gender, higher MADRS, and UPDRS—activities of daily living scores The use of DA was associated with the development of EDS
[11] Norway	Population based cohort, 8 years	232 PD cases	SSQ	The prevalence of EDS increased from 5.6% at baseline to 22.5% at 4 years and 40.8% after 8 years The 8-year prevalence of EDS was 52.4% EDS was a persistent feature in the majority of patients EDS was associated with age, gender, and the use of DA. In PD patients who have never used DA, EDS was associated with H and Y stage only
[12] USA	Prospective population based cohort, 7 years Honolulu-Asia Aging Study	3741 elderly male subjects	EDS was defined using a standardized protocol Insomnia was defined as having difficulty falling asleep or waking up far too early and not being able to go back to sleep. Men were reported as being groggy if they felt unrefreshed for ≥ 0.5 h after awakening in the morning. The prevalence of loud snoring was based on complaints from a spouse or housemate, and frequent nocturnal awakening was defined as waking up several times during the night for reasons other than the need to use the bathroom	43 men developed PD during the course of follow-up (19.9/10,000 person-years) Men with EDS had an excess risk of PD compared to men without EDS (OR 3.3; 95% CI 1.4 to 7.0; *p* = 0.004) Insomnia, daytime napping, early morning grogginess, and frequent nocturnal awakening were not associated with increased PD risk
[13] Germany	Prospective cohort study, 1 year	111 PD	PDQ-39: sleep problems, daytime sleepiness, bad dreams, and hallucinations	Sleep problems were reported by 1/3 in PD subjects with and without DA EDS was higher in the two DA groups (ergoline 11.9%, non-ergoline 9.1%) than among patients not taking Das (4.5%) Sleep problems decreased among those taking DA continuously and those newly taking DA; but increased in patients discontinuing DAs EDS increased to 25% in patients newly taking DA; and decreased to 15.9% in those taking them continuously
Insomnia				
[14] PPMI cohort	Prospective cohort, 6 months	Early stage, medication naïve PD patients	STAI, GDS-15, Score ≥ 2 on item 1.7 of the MDS-UPDRS	Baseline insomnia in PD was not associated with GDS-15 or STAI total scores at follow-up Insomnia predicted higher STAI state scores Baseline STAI scores and GDS-15 score were significantly associated with insomnia at follow-up
[15] The Netherlands	Prospective cohort, 5 years	421 PD subjects	SCOPA-SLEEP-Nighttime sleep (NS) problems section Insomnia was defined as scores ≥ 7	At baseline, 27% of PD subjects had insomnia For the remainder of subjects, 33% developed insomnia at some point during follow-up Higher SCOPA-SLEEP-NS scores were associated with more severe depressive symptoms, motor fluctuations, higher DA doses, and sleep medication use
[16] Norway	Population-based incident cohort, 5 years	182 drug naïve PD subjects and 202 HCs	SSQ, PDSS	The prevalence of insomnia in PD was not higher than HCs at 5 years In PD, the prevalence of solitary problems in sleep maintenance increased from 31% at baseline to 49% after 1 year; and were associated with DA use and higher MASDR scores In PD, the prevalence of solitary sleep initiation problems decreased from 21% at baseline to 7.4% after 5 years
[17] Norway	Population based, 8 years	231 PD	Semi-structured interviews	58.9% of PD subjects reported insomnia at baseline, 53.5% reported insomnia at 4 years, and 56.2% reported insomnia at 8 years The frequency of problems with sleep initiation ranged from 23 to 30%; and the frequency of frequent awakenings ranged from 23 to 44% 33% reported insomnia at all 3 visits, 30% experienced insomnia only at baseline or at baseline and at one more visit 20% developed insomnia during the study period The presence of insomnia was related to disease duration (OR 1.07, 95% CI 1.02 to 1.13, *p* = 0.009), higher MADRS scores (OR 1.06, 95% CI 1.01 to 1.11, *p* = 0.03), and the female sex (OR 1.06, 95% CI 1.01 to 1.11, *p* = 0.03)
Obstructive sleep apnea				
[18] Canada	Prospective cohort, 1 year	67 PD patients	vPSG	Baseline motor UPDRS scores were higher in PD patients with OSA compared with patients without OSA (13.6 versus 16.2, *p* < 0.001) CPAP use was associated with stabilization of motor function (mUPDRS and timed up and go (TUG) testing) over time
[19] Taiwan	Retrospective cohort, 3 years National Health Insurance Research Database	1994 patients with OSA, 9720 matched cohort	ICD-9-CM for OSA	Over 3 years, 17 (0.9%) of the OSA cohort and 38 (0.4%) of the matched cohort developed PD The OSA cohort had a 1.85 higher risk of PD (95% VI = 1.02–3.35), *p* = 0.042) compared to the control cohort; this was seen only in males (adjusted HR 2.26; 95% CI = 1.11–4.63; *p* < 0.05) and those aged ≥ 60 years (adjusted HR, 1.93; 95% CI = 1.01–3.71; *p* < 0.05)
[20] Taiwan	Retrospective cohort, 5.6 years Taiwan Longitudinal Health Insurance Database	16,730 newly diagnosed OSA, 16,730 matched controls from a random sample of 1 million individuals	ICD-9-CM codes for OSA	Patients with OSA were 1.37 times more likely to have PD than patients without OSA (95% CI = 1.12–1.68, *p* < 0.05) Subgroup analysis showed that patients with coronary artery disease, stroke, or chronic kidney disease had a higher risk of PD
[21] Taiwan	Retrospective longitudinal cohort, 5 years Taiwan Longitudinal Health Insurance Database	1532 patients with a first-time diagnosis of OSA, random control group of 7660 patients	The presence of OSA was diagnosed by the ICD-9-CM codes	In the 5-year follow-up period, PD developed in 1.24% of the OSA group and 0.73% in the control group For patients with OSA, the HR for developing PD over 5 years was 2.26 (95% CI = 1.32–3.88) compared with controls In females with OSA, the HR for PD was 3.54 (95% CI = 1.50–8.34) for patients with OSA compared to patients without OSA In males with OSA, there was no significant increased hazard of PD compared to those without OSA
[22] Taiwan	Retrospective longitudinal cohort, 11 years Taiwan Longitudinal Health Insurance Database	5864 patients with OSA, 23,269 control subjects without OSA	ICD-9-CM code for OSA	The incidence of PD in the OSA cohort was 2.56 per 1000 person-years compared to 1.84 per 1000 years in the control cohort The risk of PD was greater in subjects with both OSA and insomnia (adjusted HR 1.97, 95% CI 1.44–2.69) than for the control cohort Females with OSA aged 50–69 years had the highest risk for PD (adjusted HR = 2.82)
REM sleep behavioral disorder				
*Association of RBD with the development of Parkinson’s Disease*				
[23••] International RBD study group	Prospective cohort, follow-up duration between 1 and 19 years, average 4.6 years	1280 idiopathic RBD subjects	vPSG	The overall conversion rate from iRBD to an overt neurodegenerative syndrome was 6.3%/year, with 73.5% converting after 12-year follow-up Risk factors for conversion included abnormal quantitative motor testing (HR = 3.16), objective motor examination (HR = 3.03), olfactory deficit (HR = 2.62), mild cognitive impairment (HR = 1.91–2.37), erectile dysfunction (HR = 2.13), motor symptoms (HR = 2.11), an abnormal DAT scan (HR = 1.98), color vision abnormalities (HR = 1.69), constipation (HR = 1.67), REM atonia loss (HR = 1.54), and age (HR = 1.54)
[24] US	Retrospective cohort, 16 years	26 idiopathic RBD subjects	vPSG	80.8% of idiopathic RBD patients developed parkinsonism or dementia during the 16-year follow-up. The mean interval from iRBD onset to parkinsonism/dementia onset was 14.2 ± 6.2 years
[25] Canada	Retrospective cohort, of all subjects diagnosed with RBD between 1989 and 2006	93 idiopathic RBD patients	vPSG	26/93 subjects developed a neurodegenerative disorder during the period of follow-up The 5-year risk of neurodegenerative disease was 17.7%, the 10-year risk 40.6%, and the 12-year risk was 52.4%
[26] Spain	Retrospective cohort, 5.1 years	44 idiopathic RBD patients	vPSG	45% of patients developed a neurological disorder after a mean of 11.5 years from the reported onset of RBD Patients with a longer clinical follow-up developed neurological disease (OR 1.512, 95% CI, 1.105–2.069, *p* = 0.010)
[27] US	Retrospective cohort, 6.1 ± 2.4 years	29 idiopathic RBD patients	vPSG	38% of idiopathic RBD patients developed a parkinsonian disorder over the follow-up period, at a mean interval of 12.7 ± 7.3 years after the onset of RBD
*Longitudinal studies with vPSG*				
[28] Germany	Prospective cohort 6 years	98 de novo PD	vPSG	In PD, RBD prevalence increased from 25% at baseline to 52% after 6 years RWA increased from 19% at baseline to 41% after 6 years; growing by 29.7% every 2 years (*p* < 0.001) Time (p < 0.001) and age (p < 0.001) were independent factors influencing RWA increase. There was no effect of sex, LEDD, UPDRS total scores, benzodiazepines, MMSE and dyskinesia on RWA
[29] France	Prospective cohort, 3 years	22 moderate to advanced disease PD	vPSG, self-reported symptoms of RBD evaluated using clinical interview	Self- assessed frequency of RBD could increase (6/22), decrease (6/22) or remained stable (10/22) over 3 years RWSA measures increased significantly in all subjects; and was correlated with dyskinesias (*r* = 0.61, *p* = 0.05), motor fluctuation (*r* = 0.54, *p* = 0.03), worsening executive function (*r* = 0.78, *p* = 0.001), and visuo-spatial perception (*r* = − 0.57, *p* = 0.04)
[30••] Germany	Prospective cohort, 2 years	113 de novo PD and 102 HCs	vPSG	The prevalence of behaviors during REM sleep in PD increased from 50 to 63% after 2 years (*p* = 0.02) In PD, the prevalence of RBD increased from 25 to 43% after 2 years (*p* < 0.001) 38% of RBE not yet fulfilling diagnostic criteria for RBD and 18% of PD patients with normal REM sleep converted to RBD after 2 years In HC, the prevalence of behaviors during REM sleep increased from 17 to 20% (n.s.); the prevalence of RBD increased from 2 to 4% (n.s.) 7% of RBE positive HC and 1% of HC with normal REM at baseline converted to RBD
*Prevalence studies of RBD without the use of vPSG*				
[31] Denmark	Prospective cohort, 3 years	96 PD patients with a disease duration of 5 years	RBDSQ	The prevalence of pRBD was 38% at baseline, which increased to 49% after 5 years The pRBD status was unchanged in ¾ of the cohort
[32] Portugal	Prospective cohort, 2 years	61 early-stage PD	RBDSQ clinical interview	The prevalence of RBD increased from 25/61 at baseline to 35/61 after 2 years 3 subjects with RBD reverted to non-RBD at follow-up, while 10 subjects developed RBD at follow-up. The annual incidence of RBD is 12.5% The presence of RBD at baseline was associated with increases in UPDRS total and bradykinesia scores over time
[33] France	Prospective cohort, 2 years	61 PD	Clinical interview with the spouse	64% had RBD at baseline, and 52% had RBD 2 years later. The annual incidence rate of clinical RBD onset was 9% and disappeared at a rate of 14% per year Patients with clinical RBD had earlier disability than those without RBD, but there was no specific worsening of both motor and non-motor symptoms in subjects with clinical RBD compared to those without
[34] Norway	Prospective, population-based cohort, 8 years	231 PD	Sleep questionnaire to determine the presence of pRBD	The prevalence of pRBD in PD could vary across the duration of follow-up: 14.7% at baseline, 27.3% at 4 years, and 14.6% at 8 years pRBD was associated with the male gender, less parkinsonism, and higher LEDD
*Studies predominantly looking at the effect of RBD on prognosis of PD*				
[6] UK	Prospective cohort, 4.8 ± 1.98 years	923 early PD (diagnosed within 3.5 years)	RBDSQ	33.3% of PD subjects were identified as having pRBD at baseline The presence of pRBD was associated with more severe baseline symptoms and faster progression on the MDS-UPDRS part 1 and 3, Purdue Pegboard test, BDI scores The presence of pRBD was associated with an increased risk of mild cognitive impairment (HR = 1.36, 95% CI 1.01–1.83), freezing of gait (HR –1.42, 96% CI 1.10–1.85), and frequent falls (HR = 1.62, 95% CI, 1.02–2.60)
[35] UK	Prospective cohort, 1 year	921 early PD within 3.5 years of diagnosis, HCs	RBDSQ	The presence of RBD at baseline was not associated with an increased risk of development impulse control disorders
[36] Canada	Prospective cohort, 4 years	42 non-demented PD subjects	vPSG	48% of PD subjects with RBD developed dementia after 4 years; none of the PD subjects without RBD developed dementia after 4 years (*p* < 0.014) Baseline RSWA predicted development of dementia (% tonic REM = 73.2 ± 26.7 with dementia, 40.8 ± 34.5 without dementia; *p* = 0.029) The presence of RBD at baseline predicted the new development of hallucinations and cognitive fluctuations
[37] Italy	Prospective cohort, 2 years	3 groups of PD patients: 27 without RBD or hallucinations, 32 with RBD only 40 with RBD and hallucinations	Clinical diagnosis of RBD	After 2 years, subjects without RBD and hallucinations had normal neuropsychological assessments and remained free of RBD and hallucinations After 2 years, approximately 1/3 of subjects with RBD only developed hallucinations After 2 years, subjects with both RBD and hallucinations at baseline had cognitive worsening and a higher mortality rate
[38] Italy	Prospective cohort, 8 years	80 PD patients	Sleep questionnaire to reflect the ICSD diagnostic criteria for RBD; vPSG was performed in patients thought to have RBD	The presence of RBD was significantly related to the development of hallucinations, independent of age, gender, or duration but dependent on the amount of dopaminergic therapies
Restless legs syndrome				
[39] Germany	Prospective cohort, 4 years	135 de novo PD and 109 HCs	vPSG for 2 consecutive nights	PLMD did not differ between de novo PD and HCs at baseline (32.83 versus 27.72, *p* = 0.0297) PLMD was one of the predictors of cognitive impairment in PD
[40] US	(Historic) Prospective population cohort, case control study, 7.8 years	100,882 PD free subjects: 58,475 with prevalence RLS and 58,475 propensity-matched cohort were extracted from the veterans database consisting of 3.5 million veterans	ICD-9-CM code for restless legs syndrome	68 incident PD events (0.13%, incidence rate 1.87 [1.48–2.37]/10,000 patient-years) occurred in the RLS negative group 185 incident PD events (0.37%, incidence rate 4.72 [4.09–5.45]/10,000 patient-years) occurred in the RLS-positive group in the propensity-matched cohort Prevalent RLS was associated with a higher risk of incident PD (HR 2.57, 95% CI 1.95–3.39) compared to RLS-negative patients
[41] Italy	Prospective cohort, 4 years	109 newly diagnosed, de novo PD	RLS Diagnostic Index	In PD, the prevalence of RLS increased from 4.6% at baseline to 6.5% after 2 years to 16.3% after 4 years (*p* = 0.007) At baseline, RLS was more likely to be associated with insomnia (OR = 15.555, *p* = 0.040) At follow-up, RLS was more likely to be associated with dizziness (OR = 1.153; *p* = 0.022) and daytime sleepiness (OR = 9.557, *p* = 0.001) compared to patients without RLS Older age was more likely associated to increased RLS occurrence (OR = 1.187, *p* = 0.036) Increased dopaminergic transporter availability of the affected caudate and putamen was more likely to be associated with RLS presence at diagnosis (OR 75.711, *p* = 0.077) and RLS occurrence (OR = 12.004, *p* = 0.059) as compared to patients without RLS (*n* = 88)
[42] USA	Prospective cohort, 8 years	22,999 male subjects from the Health Professionals Follow-up Study	3 questions based on the IRLSSG criteria	200 incident PD cases were identified during 8 years of follow-up Men with RLS with symptoms greater than 15 times/month had a higher risk of PD development (adjusted relative risk = 1.47; 95% CI 0.59, 3.65; *p* = 0.41) However, this was statistically significant only for cases diagnosed within 4 years of follow-up (adjusted relative risk = 2.77; 95% CI 1.08, 7.11; *p* = 0.03). This was not statistically significant across the entire 8-year period of follow-up
[43] Italy	Prospective cohort, median 26 months	106 de novo PD patients,	IRLSSG criteria	Over the study period of 26 months, 15 subjects developed RLS; giving an incidence rate of total RLS was 47 per 1000 case/person per year and 37 per 1000 case/person per year after exclusion of secondary causes of RLS 83.3% developed RLS within 24 months from the starting of dopaminergic therapy, with a median latency of 7.5 months
Shift work/sleep duration				
[44] USA	Prospective study, 12 years follow-up	84, 794 female nurses US Nurses’ Health Study	Self-reported duration of rotating night shifts and sleep duration	Nurses with 15 years or more of night shift work had a 50% lower risk of PD compared to those who never worked night shifts. (95% CI 0.26, 0.97; *p* = 0.01) Increased sleep duration was associated with increased PD risk: comparing nurses who sleep > 9 h per day with those who slept ≤ 6 h, the RR for PD was 1.84 (95% CI 0.99, 3.42, *p* = 0.005) when comparing nurses who reported 9 or more hours of sleep per day with those who slept 6 h or less (*p*(trend) = 0.005)
General sleep complaints or multiple sleep disorders				
[45] Portugal	Prospective cohort, 4 years	25 PD	vPSG, SCOPA-SLEEP daytime and nighttime	Lower N3% at baseline was significantly associated with decreases in MOCA scores over time Higher PLMD was associated with SCOPA daytime score increases over time RBD was significantly associated with SCOPA daytime score increases over time Higher global severity of RBD, tonic RWSA, and total number of motor events during REM sleep were associated with SCOPA-nighttime score increases
[46] Portugal	Prospective cohort, 3.5 years	49 PD, 20 DLB	vPSG	No significant interactions were found in vPSG data and GDS variation for the entire group and DLB separately In PD, there was a significant interaction between RBD diagnosis, tonic excessive muscular tone, and GDS increase
[47] China	Prospective cohort, 3 years	224 early-stage PD	Sleep/fatigue subscore of the NMSS	Increases in the sleep/fatigue subscore of the NMSS was observed from baseline at 1 year, 2 years, and 3 years, with a relative change of − 1.51%, 14.87%, and 19.46%, respectively However, the overall effect size from baseline to 1 year, 2 years, and 3 years were very small, at 0.01, − 014, and − 0.20, respectively
[48] Spain	Prospective cohort, follow-up range from 8 to 125 months with variable mean follow-up duration of 48.5 ± 26.6 months	103 PD, disease stage ranged from early-stage to advanced disease	PDSS, ESS	The baseline prevalence of nocturnal sleep disturbances was 48.5% and EDS was 40% Slight improvements in PDSS and ESS scores were observed over time Nocturnal sleep problems were associated with age and motor impairment Higher ESS scores were associated with higher motor impairment, higher combined treatment doses of levodopa and DA, and lower ESS scores were associated with disease duration and L-dopa monotherapy
[49•] PPMI cohort	Prospective cohort, 5 years	218 early disease, de novo PD subjects and 102 HCs	ESS, Insomnia using the UPDRS Part I Subitem 1.7 and RBDSQ	At baseline, 31.17% of PD subjects reported one type of sleep disturbance, 11.5% reported two types of sleep disturbances, and 1.4% reported all three of sleep disturbances studied At 5 years, 39.0% of PD subjects reported one type of sleep disturbance, 23.4% reported two types of sleep disturbances, and 7.3% reported all three types of sleep disturbances The largest increase in frequency was seen in insomnia (44.5%), followed by EDS (32.1%) and pRBD (31.2%) Insomnia was the most common sleep disturbance at any time point
[50] Taiwan	Prospective cohort, average follow-up of 9.9 years National Health Insurance Research Database	91,273 adult patients with non-apnea sleep disorders, with an age, gender, income, urbanization, and Charlson comorbidity index score matched control cohort of 91,273 subjects from the Taiwan National Health Insurance Research Database	Claims data of ICD-9-CM codes of insomnia, sleep disturbance, sleep–wake schedule, and parasomnias were used to define the non-apnea sleep disturbances Subjects with ICD-9-CM codes of sleep apnea, hypersomnia with sleep apnea, unspecific sleep apnea, and PD were excluded from the analysis	Patients with non-apnea sleep disorders were more likely to develop PD (log-rank test, *p* < 0.001) on Kaplan–Meier analysis Non-apnea sleep disorders was an independent risk factor for PD (crude HR 1.63, 95% CI 1.54–1.73, *p* < 0.001; adjusted HR 1.18, 95% CI 1.11–1.26, *p* < 0.001) Subjects with chronic insomnia had the greatest risk of PD (crude HR 2.91, 95% CI 2.59–3.26, p < 0.001; adjusted HR hazard ratio 1.37, 95% CI 1.21–1.55, *p* < 0.001)
[51] PPMI	Prospective cohort, 2 years	423 de novo early stage PD, 196 HCs	UPDRS Part 1 Scores, ESS, RBDSQ	In PD subjects, there was a non-significant increase in sleep problems over 2 years (*p* = 0.0655). As a group, PD subjects reported more sleep problems compared to HCs ( *p* = 0.0010) In PD subjects, there was an increase in daytime sleepiness over 2 years (*p* < 0.0001). The prevalence of EDS in PD increased from 15.6 to 22.67% over 2 years (*p* = 0.4782) In PD subjects, the prevalence of pRBD increased from 37.86 to 41.87% *p = 0.4137)
[52] Canada	Prospective cohort, 4.5 years	68 non-demented PD, 44 HCs	vPSG	In REM sleep, PD patients who later developed dementia showed higher absolute power in delta and theta bands and a higher slowing ratio compared to patients who remained dementia free or controls In NREM sleep, lower baseline sigma power in the parietal cortical regions predicted the development of dementia During wakefulness, lower dominant occipital frequency and higher delta and slowing ratio were seen in PD patients who later developed dementia, compared to those who remained dementia free and controls Overall, REM sleep slowing ratios in the posterior regions, wakefulness slowing ratios in the temporal regions, and lower dominant occipital frequency best predicted dementia
[53] Canada	Prospective cohort, 4.5 years	68 non-demented PD, 47 HCs	vPSG	Sleep spindle density and amplitude were lower in PD patients who developed dementia compared with both PD patients who remained dementia free and controls Dementia-free PD subjects had lower baseline sleep spindle density compared with controls (*p* < 0.01) Lower sleep spindle amplitude in the parietal and occipital areas were associated with poorer visuospatial abilities in demented PD patients PD patients had lower slow wave amplitude compared with HCs (*p* < 0.0001), but no difference was observed in slow wave sleep amplitude in those who developed or did not develop dementia
[54] Italy	Prospective cohort, 4 years	61 de novo, early stage PD	NMSQuest-sleep domain – daytime sleepiness, insomnia, vivid dreams, acting out, restless legs	The percentage change (4 versus 2 years) for the different sleep disturbances was 290.3% for daytime sleepiness (*p* < 0.01), 12.2% for insomnia, 113.6% for vivid dreams (*p* < 0.01), no change for acting out, and 150.7% for restless legs
[55] Italy	Prospective cohort, 2 years	91 de novo, early stage PD	NMSQuest	The percentage change (2 years versus baseline) for the different sleep disturbances was all non-significant (*p* > 0.05): 33.3% for daytime sleepiness, 4.7% for insomnia, 50% for intense, vivid dreams, + 14.8% for acting out dreams, and − 33.3% for restless legs
[10] Norway	Population based, incident cohort, 1 year	138 drug-naïve, early PD and 138 HCs	PDSS	Total PDSS scores for PD patients were lower for patients compared with controls, 119 vs 127 (*p* < 0.05), at baseline and 121 versus 128 (*p* < 0.005) after 1 year on drugs PD patients had higher nocturnal motor off symptoms and increased daytime somnolence at baseline and 1 year compared with HCs Only minor changes in sleep scores were seen after introduction of dopaminergic medications
[2] USA	Prospective cohort, 10 years	89 PD patients	Modified version of the PSQI to identify subjects with acting out dreams, sleep fragmentation, and vivid dreams/nightmares	The prevalence of acting out dreams increased over time (*p* = 0.001) The presence of vivid dreams/nightmares; sleep fragmentation did not increase over time Severe sleep fragmentation (OR 2.01, *p* = 0.006) and vivid dreams/nightmares (OR = 2.38, *p* = 0.0004) were associated with concurrent hallucinations In PD subjects who did not hallucinate at baseline, sleep abnormalities at study entry did not predict future development of hallucinations
[56] USA	Prospective cohort, 6 years	38 PD patients	Modified version of the PSQI to identify subjects with acting out dreams, sleep fragmentation, and vivid dreams/nightmares	Sleep disorders fluctuated widely among patients and tie points, with no evidence of progression in severity (*p* = 0.73) The presence of vivid dreams/nightmares did not predict future development of hallucinations (OR = 0.94, *p* = 0.51)

*BDI* Beck Depression Inventory, *CSF* cerebrospinal fluid, *CI* confidence interval, *CPAP* continuous positive airway pressure therapy, *DA* dopamine agonists, *DAT* dopamine transporter imaging, *DLB* dementia with Lewy bodies, *ESS* Epworth Sleepiness Scale, *EDS* excessive daytime sleepiness, *GDS* global deterioration scale, *GDS-15* 15-item Geriatric Depression Scale, *HCs* health controls, *H and Y stage* Hoehn and Yahr stage, *HR* hazard ratio, *iRBD* idiopathic rapid eye movement sleep behavior disorder, *ICD-9-CM* International Classification of Disease, 9th edition, Clinical Modification, *ICSD* International Classification of Sleep Disorders, *IRLSSG* International Restless Legs Syndrome Study Group Criteria, *LEDD* levodopa equivalent dose, *MADRS* Montgomery-Asberg Depression Rating Scale, *MDS-UPDRS* Movement Disorders Society – Unified Parkinson’s Disease Rating Scale, *OR* odds ratio, *MMSE* mini-mental state examination, *NMSQuest* non-motor symptoms questionnaire, *NMSS* non-motor symptoms scale, *NREM* non-rapid eye movement, *N.s.* not significant, *PD* Parkinson’s disease, *OSA* obstructive sleep apnea, *PDQ 39* Parkinson’s Disease Questionnaire, *PDSS* Parkinson’s Disease Sleep Scale, *PLMD* periodic limb movement index during sleep, *PPMI* Parkinson’s Progression Markers Initiative, *pRBD* probable REM sleep behavior disorder, *PSQI* Pittsburgh Sleep Quality Index, *RBE* REM behavioral events not meeting criteria for RBD, *RBD* rapid eye movement sleep behavior disorder, *RBDSQ* REM sleep behavior screening questionnaire, *REM* rapid eye movement, *RSWA* REM sleep without atonia, *RLS* restless legs syndrome, *RWA* REM sleep without atonia, *SCOPA* scales for outcomes in Parkinson’s disease, *SSQ* Stavanger Sleepiness Questionnaires, *STAI* State-Trait Anxiety Inventory, *vPSG* videopolysomnography

Generally, sleep disturbances in PD patients have been studied in isolation, in the context of a broad range of non-motor symptoms, in cross-sectional or in short-longitudinal cohorts. Only very few studies have examined the frequency and coexistence of multiple sleep disorders in individual patients, and their longitudinal changes over time. Therefore, in the sections below, we have first described the longitudinal studies performed on individual sleep disturbances. We have then described the studies that have assessed the coexistence of multiple sleep problems in individual patients. Finally, we have reported some studies that have shown how vPSG features could be predictive of development of dementia in PD patients.

## Excessive Daytime Sleepiness

EDS is defined as “the inability to stay awake and alert during the major waking episodes of the day, resulting in periods of irrepressible need for sleep or unintended lapses into drowsiness or sleep” [57]. In PD, most studies define EDS using the well-established, easily performed subjective Epworth Sleepiness Scale (ESS) questionnaire [58], rather than the objective mean sleep latency test (MSLT) [59]. The MSLT provides objective measures of sleepiness on a single day, but does not reflect a subject’s average experience of sleepiness across a range of daily activities which the ESS attempts to quantify [60]. However, EDS is a symptom, with many possible causes including obstructive sleep apnea (OSA) which requires a respiratory sleep study or videopolysomnography (vPSG) for diagnosis. This is infrequently performed in longitudinal studies studying EDS in PD.

An epidemiological study from the Honolulu-Asia Aging study studying elderly male subjects showed that EDS was associated with an almost threefold increase in the risk of PD compared to subjects without EDS [12]. Other sleep features studied included insomnia, daytime napping, early morning grogginess, and frequent nocturnal awakening that were not associated with increased PD risk.

Six longitudinal studies have been performed investigating EDS in PD. In an earlier prospective cohort studied over a year, dopamine agonists (DA) were particularly implicated in causing sedation [13]. However, this finding has not been consistent across all studies with one study showing that DA did not alter EDS [10].

Subsequent longitudinal studies have focused on the prevalence of EDS in PD and its change over time: EDS was shown to be present at baseline [8, 9], with most studies reporting increased prevalence of EDS [8, 9, 11] or increase in ESS scores over time [6]. Only one small study of 30 moderate stage PD subjects showed that ESS scores remained stable over 10 years [7] and also highlighted variability of the ESS scores. Other larger studies, however, have shown that EDS is a persistent and progressive feature in most patients [11].

Across the various studies, EDS was typically associated with non-motor features such as poor sleep quality, fatigue, anxiety, depression or axial, postural, or gait disturbances rather than motor severity [7, 10, 61], or cognitive dysfunction [61]. Other studies have shown an association between EDS with greater motor severity and male gender [10, 11].

## Insomnia

“Insomnia Disorder” is defined as the persistent difficulty with sleep initiation, sleep maintenance, or quality in an individual with adequate opportunity for sleep, resulting in daytime impact. It must be present for > 3 months and occur most days of the week [57]. Hence, the diagnosis is dependent on the subjective self-reporting of symptoms and the patient’s perspective of impact on daytime function. Existing longitudinal studies of insomnia in PD have utilized different methodologies to ascertain the presence of insomnia, ranging from semi-structured interviews to standardized questionnaires, and have reported conflicting results.

The overall prevalence of insomnia in two longitudinal cohorts was not shown to change over time [16, 17] but another study showed a slight increase in the prevalence of insomnia in a cohort studied over 5 years [15]. Significant fluctuation in the reported symptoms of insomnia over time was observed in a PD cohort studied over 8 years [17]: with the proportion of subjects having difficulties with sleep initiation or frequent awakenings increasing over time. Another group did not show an increase in prevalence of insomnia over time, but showed changes in the prevalence of insomnia subtypes: with the prevalence of solitary problems in sleep maintenance increasing in the first year but the prevalence of solitary sleep initiation problems decreasing over 5 years [16].

Epidemiological studies in the general population have established that the presence of insomnia or poor sleep quality results in an increased lifetime risk of depression; with females more likely than men to report insomnia in every age group [62], this is unsurprising as the same associations are well described in the PD population. Studies of insomnia in PD have been consistent in the association of insomnia with mood disorders: one study showed that baseline anxiety and depression predicted the development of insomnia in early PD [14]. However, unlike findings from the general population, the presence of insomnia at baseline did not predict subsequent affective symptoms. This discrepancy could be due to the shorter length of follow-up in the PD population studies compared to epidemiological studies performed in the general population. Other earlier mentioned studies have shown the association of insomnia with PD disease duration [17], depressive symptoms [15, 17], female gender [17], motor fluctuations [15], and higher LEDD use [15].

## Obstructive Sleep Apnea

OSA is a common sleep disorder characterized by snoring and recurrent collapse of the airways during sleep leading to unrefreshing night sleep and daytime sleepiness [63]. It is diagnosed on respiratory polygraphy or vPSG by the presence of apneas and hypopneas during sleep [63]. Risk factors include increasing age, male gender, and obesity and the incidence is rising across the western world [63].

As OSA results in hypoxia, inflammation, and oxidative stress [64], its role as a prospective risk factor for the development of PD has been of recent interest and studied by four different groups using data obtained from the Taiwan Longitudinal Health Insurance Database [19–22]. All studies reported that the presence of OSA was associated with an increased risk of PD (OR 1.35 to 3.54), differing according to the specific subgroup studied. These studies utilizing the Taiwan Longitudinal Health Insurance Database identified subjects with OSA using the International Classification of Disease, 9th edition, Clinical Modification (ICD-9-CM) codes for OSA. Polysomnography data, in particular the apnea–hypopnea index (AHI), were not available.

Although very large study numbers were available over 3 to 11 years, there were low absolute numbers of incident PD. The hazard ratio for the risk of PD was slightly elevated, suggesting that OSA is not a particularly strong risk factor for the development of PD. Further subgroup analyses showed that OSA in combination with coronary artery disease [20], stroke [20], chronic kidney disease [20], and insomnia [22] could increase the risk of PD. However, conflicting data with regard to increased risk for PD according to gender was found: one study showed that the increased risk of PD was only observed in males with OSA [19], while a different study did not find this association but showed that in females with OSA, the HR for PD was elevated at 3.54 [21]. Finally, another study with data over 11 years showed that females subjects aged 50–69 years with OSA had the highest risk for PD [22]. However, these findings have not yet been replicated in other study populations.

The symptoms of OSA are effectively treated with continuous positive airway pressure (CPAP) therapy. However, the effects of CPAP treatment on cardiovascular events and cognitive functioning in the general population remain uncertain requiring further study with larger randomized cohorts [63]. In a recent prospective cohort of 67 PD subjects followed up for a year, CPAP therapy was shown to stabilize motor function as compared to other patient groups who did not have CPAP therapy or were not diagnosed with OSA [18]. However, this cohort was small, and replication in future studies will be necessary.

## REM Sleep Behavior Disorder

It is now well established that “isolated” RBD is a prodromal stage before overt neurodegeneration. In a landmark, multicentre study that recruited 1280 cases of vPSG confirmed RBD followed up prospectively for an average of 4.6 years, phenoconversion to a neurodegenerative disorder occurred at a rate of 6.3%/ year, with up to 73.5% phenoconverting when followed up to 12 years from RBD onset [23••]. Earlier retrospective longitudinal series of vPSG-confirmed idiopathic RBD cases have all demonstrated a high phenoconversion rate of RBD to a neurodegenerative disorder [24–27]. Schenck et al. followed up subjects to 16 years and showed the development of a neurodegenerative disorder in 80.8% of their series. A recent multimodal imaging study of RBD has proven that subjects with RBD already have established abnormalities in the peripheral autonomic nervous system and locus coeruleus, which were equivalent to that observed in PD [65].

Rather than using vPSG which is the gold standard required for RBD diagnosis, most longitudinal cohort studies in PD have instead utilized screening questionnaires such as the RBD screening questionnaire (RBDSQ) [66]. The RBDSQ has a sensitivity of 0.96 in sleep clinic cohorts, and a specificity that increases to 0.92 in unselected control groups from the general population [66]. The questionnaire has since been validated for use in PD. More recent studies using the RBDSQ to assess for the presence of RBD have shown that the prevalence of RBD in PD increases over time [31, 32], but these studies differed on whether RBD was a persistent feature [31] or could fluctuate over time [32]. Earlier studies utilizing less robust methodologies such as clinical interview with the spouse [33] and an unvalidated questionnaire [34] have suggested that RBD could fluctuate over time. This is also reflected in a large meta-analysis of eight studies in newly diagnosed PD (*n* = 2462), which reported a wide range of reported prevalence (4.3 to 69.4%) of RBD across the different studies [67]. Only one study had utilized vPSG and reported a baseline prevalence of RBD of 25% at baseline [30••]. 

These earlier studies in PD which have not studied RBD with the vPSG have since been superseded by studies utilizing vPSG at multiple time points and have conclusively demonstrated that RBD, which is characterized by REM sleep without atonia (RSWA), is a persistent and progressive feature in RBD. Using vPSG, the DeNoPa study group showed that the RBD prevalence had increased from 25% at baseline to 43% at 2-year follow-up [30••] and to 52% at 6-year follow-up [28]. RSWA was independently associated with disease duration and age but not other disease-related factors, suggesting that RSWA could be a progression marker. The authors have also introduced the concept of REM behavioral events (RBE), where REM-associated minor motor behaviors and/or vocalizations were seen but the RSWA were below cut-off values. In their series, 31 subjects identified to have RBE at baseline developed full-blown RBD within 6 years [28]: 38% of RBE positive PD subjects converted to RBD, and 18% of PD subjects with normal REM sleep at baseline converted to RBD over 2 years [30••]. The prevalence of RBE had also increased over time from 50% at baseline to 63% 2 years later [30••]. The authors have proposed that RBE represents a continuum between normal REM sleep and RBD, and that RBE could be regarded as prodromal RBD.

Another group that prospectively studied 22 moderate to advanced PD subjects with RBD with vPSG performed at baseline and 3 years later also confirmed that RSWA measures increased significantly in all subjects over time [29], and was associated with worsening motor fluctuation, dyskinesias, and cognitive function over time. Findings on vPSG showing worsening of RSWA measures contrasted with self-reported frequencies of RBD, with 42% unchanged, 27% increasing, and 27% decreasing over time. Similarly, the severity of vPSG-recorded RBD behaviors showed no change in 59%, worsening in 27%, and improving in 14%. It has been suggested that vPSG-recorded RBD behaviors reflect phasic EMG activity, in contrast to RSWA measures which reflect tonic EMG activity. RSWA measures have been increasingly proposed as a reliable marker of PD progression that does not remit with the disease course [68].

In cross-sectional studies of PD, RBD is associated with a more severe disease phenotype with greater motor and non-motor burden [69]. Earlier prospective studies studied the association of RBD with hallucinations in PD, and they showed that the presence of RBD predicted the development of hallucinations [37, 38] and was also associated with cognitive worsening [37] and higher mortality [37]. These preliminary findings have been subsequently confirmed in a prospective cohort of PD subjects followed up over 4 years with RBD diagnosed on vPSG [36]: half their cohort with RBD had developed dementia after 4 years, whereas PD subjects without RBD remained dementia free. The presence of RBD at baseline was also predictive of the development of hallucinations. Baseline REM sleep atonia loss was also predictive of subsequent dementia. Another large cohort study involving 923 early PD subjects followed up over an average of 4.8 years also showed that the presence of possible RBD at baseline predicted greater non-motor, motor progression over time, and was also associated with an increased risk of developing mild cognitive impairment [6]. However, another study utilizing the RBDSQ did not find an association between RBD and the subsequent development of impulse control disorders [35].

One challenge in the use of RSWA measures as a biomarker is that vPSG protocol and analysis methods used to study RBD differ across different studies. The currently accepted clinical rules of documenting RWA on vPSG using the International Classification of Sleep Disorders-3 (ICSD-3) rules do not provide cut-offs, so there is no consensus as to the EMG activity required to define RWA [70]. A standardized protocol to diagnose RBD and identify prodromal RBD has been recently proposed [71••]. Existing criteria such as the SINBAR criteria [72] to determine RWA are technically challenging, and non-informative vPSG recordings are common. This will impact upon the estimation of the true prevalence of RBD, even when performed with gold standard vPSG. The cost and night-to-night variability of RBD motor behavior severity on vPSG also places limitations on the utility of vPSG for longitudinal assessment [73, 74].

## Restless Legs Syndrome

RLS is characterized by unpleasant sensations producing the urge to move the legs, relief with movement, typically occurring at rest, and in late evening with a well-defined circadian rhythm [75]. Periodic limb movements of sleep (PLMS) occur in 80–89% of those with RLS [76], and can be quantified using the periodic limb movement index, which is a vPSG measure. However, the occurrence of PLMS itself is common and non-specific, and can occur with no symptoms or in association with certain medications [75]. The diagnostic criteria for RLS have undergone multiple revisions: earlier criteria were designed as a research screening tool rather than a clinical diagnostic tool and were criticized for their low positive predictive values ranging from 50 to 60%, leading to excessively high prevalence estimates of RLS in some studies [75]. There have been five studies of RLS or PLM index in PD utilizing different methods of RLS ascertainment, but only one incorporated a clinical assessment to diagnose RLS [43].

Both RLS and PD are disorders resulting from dopamine dysfunction. It has been hypothesized that RLS could be a prodromal marker for PD [77]. To date, there have been two large epidemiological studies which have reported an increased risk of PD in subjects with RLS. In the Health Professionals Follow-up Study, where subjects were studied prospectively over 8 years, male subjects experiencing RLS symptoms more than 15 times a month had a higher risk of PD development (adjusted relative risk 1.47); however, this finding was only statistically significant in PD diagnosed within 4 years of follow-up [42]. Another retrospective population study using over 8 years using the Veterans database with RLS defined according to ICD-9-CM codes showed an increased risk of PD in RLS (HR 2.57) [40].

However, RLS is an extremely common disorder, it remains unclear whether the observed association of RLS in PD is a chance phenomenon, an early symptom or late complication of PD. RLS is also linked to a myriad of other diseases common in the aging population. Trenkwalder and colleagues extensively reviewed the data associating RLS with different disorders and concluded that in the case of PD, the methodology of earlier studies were poor but that an association might be possible [78].

Two longitudinal studies, assessing previously untreated PD patients, showed that the prevalence of RLS increased over time. One study with follow-up over 26 months reported an incidence rate of RLS of 47 per 1000 case/person per year with 83.3% developing RLS within 24 months from starting dopaminergic therapy with a median latency of 7.5 months [43]. Another study followed up newly diagnosed, drug-naïve PD subjects that found an increase of RLS prevalence from 6.5% at baseline to 16.3% after 4 years [41], with the authors suggesting that RLS could occur as a complication of PD. RLS was found to be associated with higher age of PD onset, preserved dopaminergic pathways, insomnia at baseline, and EDS on follow-up. However, this study found that the baseline prevalence of RLS in PD did not differ from healthy controls and does not suggest that RLS is a prodromal feature of PD.

Only one study evaluated prognostic factors in PD progression with vPSG performed at baseline: the PLMD index did not differ between de novo PD and healthy controls, but an elevated PLMD index was shown to be a significant predictor of cognitive impairment [39].

## Shift Work Disorder/Sleep Duration

The role of sleep in the clearance of amyloid from the cerebrospinal fluid via the glymphatic pathways has been recently established, which may suggest a role for sleep in the prevention of Alzheimer’s disease [79••]. However, its relevance to the pathophysiology of PD remains to be confirmed. In an earlier epidemiological study using data from the US Nurses’ Health Study, paradoxically, subjects who had worked night shifts for 15 years or more were shown to have a lower risk of PD compared to those who have never worked night shifts [44].

## Studies Studying Multiple Sleep Disorders or Complaints

One of the difficulties in the longitudinal study of the various sleep disorders in PD is that individual sleep disorders or sleep complaints do not exist in isolation, with many sleep disturbances co-existing in the individual patient; thus, the study of individual sleep complaints in isolation may lack applicable clinical relevance. Some of the sleep complaints studied may refer to a symptom complex rather than a single sleep disorder. Furthermore, nighttime sleep disorders such as OSA and RLS that cause poor nighttime sleep quality can lead to EDS, or poor daytime sleep habits such as daytime napping that further impacts upon nighttime sleep quality.

In an epidemiological studying using data from the National Health Insurance Research Database, patients with non-apnea sleep disorders were found to have a higher risk of developing PD (crude HR 1.63) [50]. When approaching research into sleep disorders in PD, there is a dilemma whether to study individual sleep disorders, or study all causes of poor sleep in the individual. A number of longitudinal studies have studied sleep in PD using more general questionnaires designed to investigate sleep holistically in the patient; other groups have taken the quantitative approach of studying sleep using vPSG to prognosticate in PD. Sleep questionnaires and vPSG measure different aspects of sleep and both have limitations and advantages, and do not necessarily correlate with each other.

Most of the studies using general sleep scales have suggested that sleep disorders do not progress in PD or change over time [2, 10, 54, 55], and could also improve [48]. One study found increases in the sleep/fatigue sub-score of the Non-Motor Symptoms Scale (NMSS); however, the overall effect size was very small [47]. Findings from these studies using general sleep scales are in contrast to other studies that used sleep questionnaires studying individual sleep disorders or vPSG-based studies. Although some of these scales have been recommended for PD, the use of more generic scales does not seem as helpful.

Our group took a different approach and looked at the burden of EDS, insomnia, and pRBD separately in the Parkinson’s Progression Markers Initiative (PPMI) cohort to determine the burden of the respective sleep disorders at an individual patient level over 5 years [49•]. Different sleep questionnaires were utilized to study the individual burden of the sleep disorders. Over time, sleep disturbance increased with more subjects reporting an increased number of sleep disturbances. We found that the frequency of insomnia increased the most, followed by EDS and pRBD. An earlier study using the PPMI cohort over a shorter duration of 2 years reported a significant increase in the prevalence of EDS but not sleep problems and RBD [51]. Interestingly, we also found that the number of subjects reporting multiple sleep disturbances also increased over time. After 5-year follow-up, only 30.3% of PD patients did not report any sleep disturbances, 39.0% PD reported one type of sleep disturbance, 23.4% two types of sleep disturbances, and 7.3% reported all three types of sleep disturbances. However, there was a large variability in the combination of different sleep problems in individual patients, suggesting that they might have different pathogenesis.

## Predictive Values of vPSG

While vPSG is considered the gold standard required for the diagnosis of sleep disorders, including RBD, this test has also shown to have prognostic potentialities. As earlier described, vPSG features seen in RBD have prognostic value in PD: tonic REM sleep atonia loss can predict dementia [36] and this was replicated in another study [45]. However, vPSG findings have prognostic significance that is not confined to those with RBD alone. The latter study had also examined the rest of the vPSG data, but it did not find any association between other vPSG features and decline that was measured using the global deterioration scale. However, the same group studied slow wave sleep in a different cohort and found that lower N3 percentage at baseline was significantly associated with decline in MOCA scores over time [46]. Other vPSG features, including lower sleep spindle density and amplitudes at baseline, were found to be predictive of dementia in PD [53]. In particular, lower sleep spindle amplitudes in the parietal and occipital areas were correlated with poorer visuospatial abilities in demented PD subjects. The same group extended their work to show that specific vPSG features in both REM and NREM sleep and wakefulness had prognostic implications in PD [52]. Higher absolute power in the delta and theta bands in REM sleep, lower sigma power in the parietal regions in NREM sleep and lower dominant occipital frequency, and higher delta and slowing ratios during wakefulness were predictive of subsequent dementia.

## Conclusion

The vast majority of prospective, longitudinal PD cohorts have instead used a variety of self-administered, subjective sleep questionnaires to study sleep disorders within a more comprehensive data collection strategy. Investigating sleep disorders is usually not the main focus of such studies, and conflicting data seen in PD studies of sleep is very likely to represent different methodologies used to determine the presence of sleep disorders. Despite these study limitations, existing longitudinal sleep disorders in PD suggest that the prevalence of sleep disorders increase over time and are associated with other disease features of PD, particularly non-motor symptoms. RBD is an established prodromal feature of PD, but it remains unclear whether other sleep disorders predict future PD. There are effective treatments for many sleep disorders, but it is unclear whether treatment of sleep disorders can alter PD symptoms and their progression or reduce the risk of future PD development. We may be underusing powerful biomarkers of brain health in neurodegeneration and failing to consider good sleep in itself as a neuroprotective therapy.

## References

[1] Bargiotas P, Schuepbach MW, Bassetti CL (2016). Sleep-wake disturbances in the premotor and early stage of Parkinson’s disease. Curr Opin Neurol.

[2] Goetz CG (2010). Hallucinations and sleep disorders in PD: ten-year prospective longitudinal study. Neurology.

[3] Sobreira-Neto MA (2017). High frequency of sleep disorders in Parkinson’s disease and its relationship with quality of life. Eur Neurol.

[4] Zoccolella S (2011). Sleep disorders and the natural history of Parkinson’s disease: the contribution of epidemiological studies. Sleep Med Rev.

[5] Hogl B (2010). Scales to assess sleep impairment in Parkinson’s disease: critique and recommendations. Mov Disord.

[6] Liu Y (2021). Longitudinal changes in Parkinson’s disease symptoms with and without rapid eye movement sleep behavior disorder: the Oxford Discovery cohort study. Mov Disord.

[7] Hoglund A (2019). A 10-year follow-up of excessive daytime sleepiness in Parkinson’s disease. Parkinsons Dis.

[8] Chahine LM, Amara AW, Videnovic A (2017). A systematic review of the literature on disorders of sleep and wakefulness in Parkinson’s disease from 2005 to 2015. Sleep Med Rev.

[9] Tholfsen LK (2015). Development of excessive daytime sleepiness in early Parkinson disease. Neurology.

[10] Stefansdottir S (2012). Subjective sleep problems in patients with early Parkinson’s disease. Eur J Neurol.

[11] Gjerstad MD (2006). Excessive daytime sleepiness in Parkinson disease: is it the drugs or the disease?. Neurology.

[12] Abbott RD (2005). Excessive daytime sleepiness and subsequent development of Parkinson disease. Neurology.

[13] Happe S, Berger K, FS Investigators (2001). The association of dopamine agonists with daytime sleepiness, sleep problems and quality of life in patients with Parkinson’s disease–a prospective study. J Neurol.

[14] Rutten S (2017). The bidirectional longitudinal relationship between insomnia, depression and anxiety in patients with early-stage, medication-naive Parkinson’s disease. Parkinsonism Relat Disord.

[15] Zhu K, Van Hilten JJ, Marinus J (2016). The course of insomnia in Parkinson’s disease. Parkinsonism Relat Disord.

[16] Tholfsen LK (2017). Changes in insomnia subtypes in early Parkinson disease. Neurology.

[17] Gjerstad MD (2007). Insomnia in Parkinson’s disease: frequency and progression over time. J Neurol Neurosurg Psychiatry.

[18] Meng L (2020). Obstructive sleep apnea, CPAP therapy and Parkinson’s disease motor function: a longitudinal study. Parkinsonism Relat Disord.

[19] Chou PS (2017). Sleep apnea and the subsequent risk of Parkinson’s disease: a 3-year nationwide population-based study. Neuropsychiatr Dis Treat.

[20] Yeh NC (2016). Increased risk of Parkinson’s disease in patients with obstructive sleep apnea: a population-based, propensity score-matched, longitudinal follow-up study. Medicine (Baltimore).

[21] Sheu JJ (2015). A 5-year follow-up study on the relationship between obstructive sleep apnea and Parkinson disease. J Clin Sleep Med.

[22] Chen JC (2015). Obstructive sleep apnea and risk of Parkinson’s disease: a population-based cohort study. J Sleep Res.

[23] •• Postuma RB, et al. Risk and predictors of dementia and parkinsonism in idiopathic REM sleep behaviour disorder: a multicentre study. Brain 2019;142(3):744–759: 10.1093/brain/awz030. **Landmark large multicentre study involving 1280 cases of vPSG confirmed RBD recruited from sleep clinics that report a phenoconversion rate to neurodegenerative disorder occuring at a rate of 6.3%/year.** 10.1093/brain/awz030 PMC6391615 30789229

[24] Schenck CH, Boeve BF, Mahowald MW (2013). Delayed emergence of a parkinsonian disorder or dementia in 81% of older men initially diagnosed with idiopathic rapid eye movement sleep behavior disorder: a 16-year update on a previously reported series. Sleep Med.

[25] Postuma RB (2009). Quantifying the risk of neurodegenerative disease in idiopathic REM sleep behavior disorder. Neurology.

[26] Iranzo A (2006). Rapid-eye-movement sleep behaviour disorder as an early marker for a neurodegenerative disorder: a descriptive study. Lancet Neurol.

[27] Schenck CH, Bundlie SR, Mahowald MW (1996). Delayed emergence of a parkinsonian disorder in 38% of 29 older men initially diagnosed with idiopathic rapid eye movement sleep behaviour disorder. Neurology.

[28] Zimansky L (2021). Incidence and progression of rapid eye movement behavior disorder in early parkinson’s disease. Mov Disord Clin Pract.

[29] Figorilli M (2020). Does REM sleep behavior disorder change in the progression of Parkinson’s disease?. Sleep Med.

[30] •• Sixel-Doring F, et al. The evolution of REM sleep behavior disorder in early Parkinson disease. Sleep 2016;39(9):1737–42: 10.5665/sleep.6102. **Longitudinal study of PD with vPSG performed at baseline and 2 years. The authors introduce the concept of REM behavioural events, where REM-associated minor movement behaviours and/or vocalizations were observed but had RSWA values that were below cut-off values. Patients with RBE were subsequently shown to progress to overt RBD, suggesting that RBE represents a state of prodromal RBD.** 10.5665/sleep.6102 PMC4989262 27306265

[31] Bjornara KA, Dietrichs E, Toft M (2015). Longitudinal assessment of probable rapid eye movement sleep behaviour disorder in Parkinson’s disease. Eur J Neurol.

[32] Bugalho P, Viana-Baptista M (2013). REM sleep behavior disorder and motor dysfunction in Parkinson’s disease–a longitudinal study. Parkinsonism Relat Disord.

[33] Lavault S (2010). Does clinical rapid eye movement behavior disorder predict worse outcomes in Parkinson’s disease?. J Neurol.

[34] Gjerstad MD (2008). Occurrence and clinical correlates of REM sleep behaviour disorder in patients with Parkinson’s disease over time. J Neurol Neurosurg Psychiatry.

[35] Baig F (2019). Impulse control disorders in Parkinson disease and RBD: a longitudinal study of severity. Neurology.

[36] Postuma RB (2012). Rapid eye movement sleep behavior disorder and risk of dementia in Parkinson’s disease: a prospective study. Mov Disord.

[37] Sinforiani E (2008). REM behavior disorder, hallucinations and cognitive impairment in Parkinson’s disease: a two-year follow up. Mov Disord.

[38] Onofrj M (2002). Incidence of RBD and hallucination in patients affected by Parkinson’s disease: 8-year follow-up. Neurol Sci.

[39] Mollenhauer B (2019). Baseline predictors for progression 4 years after Parkinson’s disease diagnosis in the De Novo Parkinson Cohort (DeNoPa). Mov Disord.

[40] Szatmari S Jr, et al. Association of restless legs syndrome with incident Parkinson’s disease. Sleep. 2017;40(2):zsw065. 10.1093/sleep/zsw065. 10.1093/sleep/zsw065 PMC6084760 28364505

[41] Moccia M (2016). A four-year longitudinal study on restless legs syndrome in Parkinson disease. Sleep.

[42] Wong JC (2014). Restless legs syndrome: an early clinical feature of Parkinson disease in men. Sleep.

[43] Calzetti S (2014). A long-term prospective follow-up study of incident RLS in the course of chronic DAergic therapy in newly diagnosed untreated patients with Parkinson’s disease. J Neural Transm (Vienna).

[44] Chen H (2006). A prospective study of night shift work, sleep duration, and risk of Parkinson’s disease. Am J Epidemiol.

[45] Bugalho P (2021). Polysomnographic predictors of sleep, motor and cognitive dysfunction progression in Parkinson’s disease: a longitudinal study. Sleep Med.

[46] Bugalho P (2021). Objective sleep data as predictors of cognitive decline in dementia with Lewy Bodies and Parkinson’s disease. Sleep Med.

[47] Ou R (2021). Longitudinal evolution of non-motor symptoms in early Parkinson’s disease: a 3-year prospective cohort study. NPJ Parkinsons Dis.

[48] Del Pino R (2021). Clinical long-term nocturnal sleeping disturbances and excessive daytime sleepiness in Parkinson’s disease. PLoS One.

[49] • Xu Z, et al. Progression of sleep disturbances in Parkinson’s disease: a 5-year longitudinal study. J Neurol. 2021;268(1):312–320 10.1007/s00415-020-10140-x. **The authors looked at the individual complaints of probable REM sleep behaviour disorder, insomnia, and excessive daytime sleepiness in the individual subject over 5 years in the PPMI cohort. The frequency of insomnia increased the most, followed by EDS and probable RBD.** 10.1007/s00415-020-10140-x PMC7815601 32804280

[50] Hsiao YH (2017). Sleep disorders and an increased risk of Parkinson’s disease in individuals with non-apnea sleep disorders: a population-based cohort study. J Sleep Res.

[51] Simuni T (2018). Baseline prevalence and longitudinal evolution of non-motor symptoms in early Parkinson’s disease: the PPMI cohort. J Neurol Neurosurg Psychiatry.

[52] Latreille V (2016). Electroencephalographic prodromal markers of dementia across conscious states in Parkinson’s disease. Brain.

[53] Latreille V (2015). Sleep spindles in Parkinson’s disease may predict the development of dementia. Neurobiol Aging.

[54] Erro R (2016). The non-motor side of the honeymoon period of Parkinson’s disease and its relationship with quality of life: a 4-year longitudinal study. Eur J Neurol.

[55] Erro R (2013). Non-motor symptoms in early Parkinson’s disease: a 2-year follow-up study on previously untreated patients. J Neurol Neurosurg Psychiatry.

[56] Goetz CG (2005). Hallucinations and sleep disorders in PD: six-year prospective longitudinal study. Neurology.

[57] Medicine AAOS, International Classification of Sleep Disorders, 3rd Edition, in International Classification of Sleep Disorders, 3rd Edition. 2014: Darien , IL, USA 10.5664/jcsm.4050 PMC4153104 25221449

[58] Johns MW (1991). A new method for measuring daytime sleepiness: the Epworth sleepiness scale. Sleep.

[59] Thorpy MJ (1992). The clinical use of the Multiple Sleep Latency Test. The Standards of Practice Committee of the American Sleep Disorders Association. Sleep.

[60] Johns MW. Sensitivity and specificity of the multiple sleep latency test (MSLT), the maintenance of wakefulness test and the epworth sleepiness scale: failure of the MSLT as a gold standard. J Sleep Res. 2000;9(1):5–11. 10.1046/j.1365-2869.2000.00177.x. 10.1046/j.1365-2869.2000.00177.x 10733683

[61] Amara AW (2017). Longitudinal assessment of excessive daytime sleepiness in early Parkinson’s disease. J Neurol Neurosurg Psychiatry.

[62] Ford DE, Cooper-Patrick L (2001). Sleep disturbances and mood disorders: an epidemiologic perspective. Depress Anxiety.

[63] Veasey SC, Rosen IM (2019). Obstructive sleep apnea in adults. N Engl J Med.

[64] Lavie L (2008). Intermittent hypoxia: the culprit of oxidative stress, vascular inflammation and dyslipidemia in obstructive sleep apnea. Expert Rev Respir Med.

[65] Knudsen K (2018). In-vivo staging of pathology in REM sleep behaviour disorder: a multimodality imaging case-control study. Lancet Neurol.

[66] Stiasny-Kolster K (2007). The REM sleep behavior disorder screening questionnaire–a new diagnostic instrument. Mov Disord.

[67] Zhang J, Xu CY, Liu J (2017). Meta-analysis on the prevalence of REM sleep behavior disorder symptoms in Parkinson’s disease. BMC Neurol.

[68] Sringean J, et al. Rapid eye movement sleep behavior disorder and rapid eye movement sleep without atonia are more frequent in advanced versus early Parkinson’s disease. Sleep. 2021;44(9) 10.1093/sleep/zsab067. 10.1093/sleep/zsab067 33720377

[69] Postuma RB (2015). REM sleep behavior disorder and neuropathology in Parkinson’s disease. Mov Disord.

[70] Berry RB, Brooks R, Gamaldo CE, Harding SM, Lloyd RM, Marcus CL and Vaughn BV for the American Academy of Sleep Medicine. The AASM manual for the scoring of sleep and associated events: rules, terminology and technical specifications, version 2.2.

[71] •• Cesari M, et al. Video-polysomnography procedures for diagnosis of rapid eye movement sleep behavior disorder (RBD) and the identification of its prodromal stages: Guidelines from the International RBD Study Group*.* Sleep. 2021;10.1093/sleep/zsab257. **This detailed review critiques existing protocols to determine RWA on vPSG and propose a new standardized protocol to diagnose RBD on vPSG.** 10.1093/sleep/zsab257 34694408

[72] Frauscher B (2012). Normative EMG values during REM sleep for the diagnosis of REM sleep behavior disorder. Sleep.

[73] Sixel-Doring F (2011). Intraindividual variability of REM sleep behavior disorder in Parkinson’s disease: a comparative assessment using a new REM sleep behavior disorder severity scale (RBDSS) for clinical routine. J Clin Sleep Med.

[74] Zhang J (2008). Diagnosis of REM sleep behavior disorder by video-polysomnographic study: is one night enough?. Sleep.

[75] Allen RP (2014). Restless legs syndrome/Willis-Ekbom disease diagnostic criteria: updated International Restless Legs Syndrome Study Group (IRLSSG) consensus criteria–history, rationale, description, and significance. Sleep Med.

[76] Montplaisir J (1997). Clinical, polysomnographic, and genetic characteristics of restless legs syndrome: a study of 133 patients diagnosed with new standard criteria. Mov Disord.

[77] Chaudhuri KR (2006). Non-motor symptoms of Parkinson’s disease: diagnosis and management. Lancet Neurol.

[78] Trenkwalder C (2016). Restless legs syndrome associated with major diseases: a systematic review and new concept. Neurology.

[79] •• Xie L, et al. Sleep drives metabolite clearance from the adult brain. Science. 2013;342(6156):373–7 10.1126/science.1241224. **Landmark study carried out in vivo in mice that showed that disruption of sleep resulted in reduced convective clearance of beta-amyloid and other metabolites from the cerebrospinal fluid.** 10.1126/science.1241224 PMC3880190 24136970

